# Complete mitochondrial genome of the nesting Colombian Caribbean Hawksbill Turtle

**DOI:** 10.1080/23802359.2017.1292477

**Published:** 2017-02-23

**Authors:** Javier Hernández-Fernández, Gerson Beltrán-Torres, Leonardo Mariño-Ramírez

**Affiliations:** a Deparment of Natural and Environmental Science, Marine Biology Program, Faculty of Science and Engineering, Genetics, Molecular Biology and Bioinformatic Research Group –GENBIMOL, Jorge Tadeo Lozano University, Bogotá, Colombia;; b National Center for Biotechnology Information, National Library of Medicine, National Institutes of Health, Bethesda, MD, USA

**Keywords:** Testudines, eretmochelys imbricata, mitogenome, endangered turtle

## Abstract

The hawksbill turtle, *Eretmochelis imbricata* (Linnaeus, 1766), is an endangered sea turtle in Colombian Caribbean beach. In this study, we report the complete mitochondrial DNA sequences of hawksbill turtle. The entire genome comprised 16,386 base pairs, and a nucleotide frequency of T: 25.6%, C: 26.9%, A: G 35.4% and 12.1%. The mitogenome sequence of hawksbill turtle would contribute to better understand population genetics, and evolution of sea turtles. Molecule was deposited at the GenBank database under the accession number KP221806.

The hawksbill turtle, *Eretmochelys imbricata* (Linnaeus, 1766), is a marine turtle belonging to the Cheloniidae family, order testudines. Hawksbill is a specie distributed throughout the tropical and central Atlantic and Indo-Pacific region (Lutz & Musick 1997). In Colombia nests on the coast the Pacific and Atlactic oceans. Hawksbill presents a way of life very complex and specialized. To mature, reach adulthood, reproduce and complete the life cycle, they need a variety of means, including terrestrial beaches, open sea, coastal and estuarine waters (Cuevas et al. 2008). Hawksbills reach maturity after 20–40 years (Bowen & Karl 2007). Actually, the hawksbill turtle is classified ‘Critically Endangered’ (CR) (Mortimer & Donnelly 2008), with increased risk of disappearing, for its low abundance, and by the continued looting of eggs (Castaño-Mora 2002; Daza-Criado & Hernández-Fernández 2014) and intense demand for the shields used in the manufacture of handicrafts (Chacón 2009). This illegal trade has led to a drastic decline in hawksbill populations as it is believed that the size of the world population has fallen by almost 80% over the last 100 years (Meylan 1999). For these reasons, the hawksbill turtle conservation is a priority at national and global level (Trujillo 2009). Phylogenetics, phylogeographics and conservation genetics analyses have been carried out using mitochondrial DNA to generate conscience of its conservation. Blood samples from an individual of hawksbill turtle from the Don Diego beach in Tayrona National Park, Santa Marta, Colombia, were collected following the Dutton (1996) methodology. The sampled individuals are currently part of a head-starting project at the El Rodadero Aquarium and Museum (AMM El rodadero), an intermediate sector between Punta Gaira and Punta Cabeza de Negro (11°13"W, 74°14"N) in the city of Santa Marta, Department of Magdalena. The mtDNA of the hawksbill turtle was obtained by amplifying 24 DNA fragments of 800–1000 bp, and then were sanger sequenced. All sequences were assembled by means of the Geneious R6 program (Biomatters, Ltd., Auckland, New Zealand) and contigs carrying mitochondrial genes were identified against all BLASTX database. The phylogenetic tree was constructed used Geneious R6 and MEGA 5.2. (Tamura et al. 2011). The complete mitogenome of hawksbill turtle was annotated, which was 16,386 bp long, composed for 13 protein-coding genes (ND1, ND2, ND3, ND4L, ND4, ND5, ND6, COI, COII, COIII, ATP8, ATP6 and CytB), 22 tRNA, 2 rRNA (12S rRNA and 16S rRNA), and a non-coding control region (D-Loop) (Figure 1). This mitogenome has a typical order of terrestrial, freshwater and marine turtles (Drosopoulou et al. 2012; Duchene et al. 2012) and vertebrates in general (Boore 1999). 15 tRNA, 2 rRNA, and 12 protein-coding genes are encoded by the H-strand, and 7 tRNA and 1 protein-coding gene (ND6) are encoded by the L-strand. Our analysis of the hawksbill mitogenome is the first report in the Colombian Caribbean region, generating basic information for future studies at a genetic level and contributing to management and conservation plans for this species.

**Figure 1.**
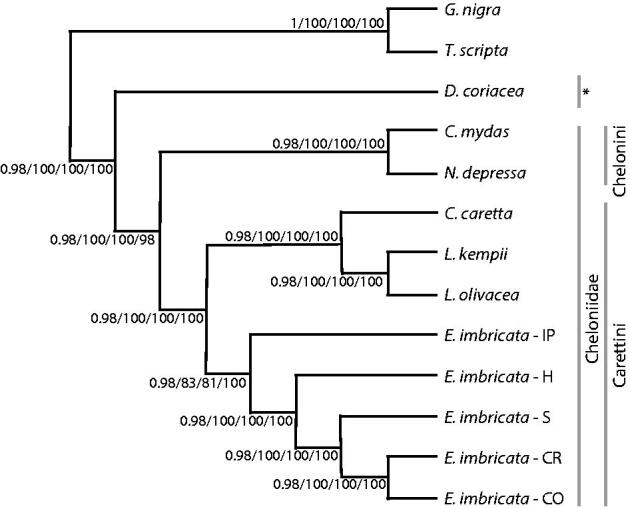
Philogenetics consensus by majority rule tree obtained using the algorithms neighbour-joining (NJ), maximum likelihood (ML), maximun parsimony (MP) and Bayesian inference (IB) used complete mitogenomes (17.384 pb). The topology of the tree shows correct association between turtles forming corresponding relationships between tribes and families. The values located in the nodes refer to the posterior probability of IB/and bootstrap, obtained with ML/MP/NJ methods. *Family Dermochelyidae.
